# Enhancing epidemic forecast usability for policymakers: A global mixed-methods study

**DOI:** 10.1371/journal.pgph.0006519

**Published:** 2026-06-04

**Authors:** Paula Christen, Loice Achieng Ombajo, Anne Cori, Jeanette Dawa, Bimandra A. Djaafara, Teresia Njoki Kimani, Camille M. J. Schneider, Sabine L. van Elsland, Mwangi Thumbi, Maria Veras, Charles Whittaker, Lilith K. Whittles, Oliver J. Watson

**Affiliations:** 1 School of Public Health, Imperial College London, London, United Kingdom; 2 Center for Epidemiological Modelling and Analysis, University of Nairobi, Nairobi, Kenya; 3 Saw Swee Hock School of Public Health, National University of Singapore and National University Health System, Singapore, Singapore; 4 Epidemiology and Modelling of Antibiotic Evasion, Institut Pasteur, Université Paris Cité, Paris, France; 5 Centre for Epidemiology and Population Health, Anti-infective Evasion and Pharmacoepidemiology team, Université Paris–Saclay, Université de Versailles Saint-Quentin-en-Yvelines, Institut national de la santé et de la recherche médicale, Montigny-Le-Bretonneux, France; 6 Paul G Allen School for Global Animal Health, Washington State University, Pullman, Washington, United States of America; 7 Faculdade de Ciências Médicas da Santa Casa de São Paulo, São Paulo, Brazil; 8 University of California, Berkeley, California, United States of America; National Center for Chronic and Noncommunicable Disease Control and Prevention, Chinese Center for Disease Control and Prevention, CHINA

## Abstract

The COVID-19 pandemic exposed critical gaps in the generation, interpretation, and use of epidemic forecasts for public health decision-making. We conducted a global mixed-methods study combining an online survey (n = 143, from 46 countries across all World Bank income groups) with 13 semi-structured interviews to examine how epidemic forecasts were perceived, used, and communicated by stakeholders involved in COVID-19 policy dialogues. Survey responses were analysed descriptively, stratified by country income group, while interview transcripts were analysed thematically using the Framework Method. Forecasts informed policy questions ranging from epidemic size estimation to intervention planning, with the projected impact of interventions (65%), epidemic peak (64%), and prevalence (62%) being the most frequently communicated metrics. Preferred formats varied by setting: 72% of high-income country (HIC) respondents valued explicit uncertainty presentation, compared with 34% in lower-middle-income countries (LMICs) and 23% in low-income countries (LICs). Barriers to forecast use were most pronounced in lower-income settings, where 47% of LIC and LMIC respondents reported that colleagues did not understand the modelling methodology, compared with 3% in HICs. Qualitative data highlighted that forecast credibility depended on interpersonal trust, institutional relationships, and contextual relevance rather than statistical sophistication alone. Findings should be interpreted in light of potential recall and selection biases inherent in retrospective, convenience-sampled designs. Strengthening forecast impact will require modular, user-oriented tools, embedding modellers within response teams, co-developing decision-relevant metrics, and sustained investment in foundational health information systems, particularly in resource-constrained settings.

## Introduction

In public health emergencies, the idealised sequence of evidence generation, appraisal, and policy application rarely holds. The COVID-19 pandemic highlighted that in crisis situations, evidence gathering, decision-making, and intervention often occur simultaneously. These overlapping processes are further complicated by compressed timelines, significant uncertainty, and highly charged political and social contexts [1].

Early in the COVID-19 pandemic, decisions were often based on incomplete data [2], past experiences (e.g., with severe acute respiratory syndrome or Middle East respiratory syndrome) [3,4], expert opinion [5], real-time international comparisons [6], and epidemiological models [7]. Epidemic forecasting, a subset of epidemiological modeling focused on short-term, probabilistic projections, emerged as a key tool for supporting decisions under uncertainty. Forecasts enabled rapid testing of policy hypotheses that would otherwise be costly, impractical, or unethical to explore [8,9]. They were developed to inform decisions across sectors and levels, from hospital resource allocation to national strategies on pharmaceutical and non-pharmaceutical interventions (NPIs) as well as international guidelines.

Since the pandemic, several frameworks have sought to strengthen the interface between modelling and policy, including the WHO-OECD-World Bank guide on integrated modelling for pandemic preparedness and response [10], and the WHO Preparedness and Resilience for Emerging Threats (PRET) initiative [11]. These initiatives underscore an emerging consensus that forecast utility depends not only on technical accuracy but on the broader ecosystem of data infrastructure, institutional trust, and knowledge translation capacity within which forecasts are produced and used.

During the pandemic, the availability and uptake of epidemiological forecasts in policy were uneven across countries and decision-making bodies [12]. These disparities were particularly pronounced in resource-constrained settings, where limited data infrastructure, scarce modelling expertise, and weak institutional linkages between researchers and policymakers constrained both the production and uptake of forecasts [13]. While efforts to build technical capacity in low- and middle-income countries are ongoing [14], strengthening expertise alone will not close the science-policy gap. In many settings, models were developed without input from policymakers or a clear understanding of the decisions they were meant to inform [15]. This disconnect between model development and the actual needs of policymakers remains a central barrier to the effective use of forecasting in public health decision-making. While aforementioned post-pandemic frameworks have begun to address the structural conditions for modelling-to-policy translation [10,16], they have largely focused on the supply side-building modelling capacity and data systems, without systematically investigating how end-users perceive, interpret, and act on forecast outputs. Understanding the demand side - what policymakers actually need from forecasts and why uptake varies across settings - remains a critical evidence gap.

To support the timely and effective use of epidemic forecasts in public health emergencies, particularly in resource-constrained settings, this study examines the types of policy questions forecasts were used to address, the metrics and formats that shaped their utility, and the unmet needs that limited their impact. Drawing on experiences from the COVID-19 pandemic, it identifies recurring patterns that influenced uptake and interpretation, with the goal of informing how forecasts can be better aligned with decision-making needs in future crises. In doing so, this work complements ongoing efforts to promote data and statistical literacy among decision-makers [17], while also calling attention to the role of the modelling community in shaping the policy relevance of their outputs [15].

## Methods

### Ethics statement

This study was conducted in compliance with the Checklist for Reporting Results of Internet E-Surveys (CHERRIES) guidelines. Ethical approval was obtained from the Imperial College Research Ethics Committee (ICREC reference number: 7095429). Participation was voluntary, and individuals could withdraw at any stage prior to data analysis. All data were pseudonymised and stored securely in accordance with institutional data protection policies. To protect participant anonymity, interview respondents were assigned numeric identifiers (e.g., ID101) and identifying details such as specific job titles, organisational names, and project-level information were removed or generalised during transcription. Survey respondents had the option to participate anonymously; those who provided contact details for follow-up were stored separately from survey responses.

### Study design

We employed a mixed-methods approach to examine the use and interpretation of epidemic forecasts in policy decision-making during the COVID-19 pandemic. A convergent design was chosen to enable complementary breadth (survey) and depth (interviews), with the quantitative component capturing patterns in forecast use across settings and the qualitative component exploring the mechanisms and contextual factors underlying these patterns. Survey reporting follows the CHERRIES guidelines, and qualitative reporting is guided by the Consolidated Criteria for Reporting Qualitative Research (COREQ). Data were collected from stakeholders involved in the development, implementation, and evaluation of pandemic response policies and programmes.

### Survey

The online survey was developed using Qualtrics software and included 24 multiple-choice and open-ended questions. Survey items were informed by a review of the literature and input from eleven experts on modeling and policy engagement. The survey consisted of five sections covering participants’ engagement in COVID-19 policy dialogues, the types, and sources of epidemic forecasts accessed, policy questions addressed using forecasts, evaluation practices, and perceived credibility of forecasts, and barriers to their effective use in decision-making (S1 Appendix A). The survey was translated into French and Spanish by native speakers and piloted with individuals experienced in modeling and policy engagement. Responses submitted in French or Spanish were translated into English for analysis. Translations were reviewed by bilingual members of the research team to ensure accuracy, though formal back-translation was not conducted.

Participants were eligible to participate in this study if they self-identified as having engaged directly or indirectly in COVID-19 policy dialogues. These dialogues were defined as decision-making processes related to the planning, development, or implementation of COVID-19 policies. Direct engagement referred to participation in meetings or discussions, while indirect engagement included the provision of evidence or technical advice to decision-makers.

Participants were recruited using convenience sampling through existing professional networks, snowball sampling, social media, and outreach during a satellite event at the Infectious Disease Modelling Conference, Bangkok, in November 2024. In addition, an email containing study information and a survey link was distributed using Microsoft Outlook Mail Merge software in December 2024. Upon clicking the link, participants were directed to a webpage containing a participant information sheet. Informed consent was obtained electronically before proceeding to the survey. The survey was open, meaning any eligible individual could participate. Participation was voluntary, and respondents had the option to remain anonymous, and to stop completing the survey at any point. The survey remained open for six months, from 23 September 2024 until 20 March 2025.

As the survey was disseminated through open channels without a defined sampling frame, a response rate cannot be calculated. We recognise that convenience and snowball sampling may introduce selection bias, particularly toward individuals with greater engagement in modelling or policy networks. To partially mitigate this, recruitment was diversified across multiple channels, professional networks, social media, a satellite conference event, and targeted email outreach, and extended to French- and Spanish-speaking respondents to broaden geographic reach beyond Anglophone networks. We also stratified analyses by World Bank income classification to assess whether patterns held across settings with differing levels of proximity to modelling communities. The six-month data collection period may also have introduced temporal variation in responses as post-pandemic contexts evolved; however, the extended timeframe was necessary to maximise geographic and professional diversity.

### Interviews

Semi-structured interviews were conducted via Microsoft Teams. Participants included a purposive sample of survey respondents who consented to follow-up, as well as additional individuals recruited specifically for the interview phase. Participants were selected to ensure diversity across regions, policy engagement types, and type of organization. Where preferred, interviews were conducted in the participant’s native language and later translated into English for analysis. Translations were reviewed by bilingual members of the research team to ensure accuracy, though formal back-translation was not conducted. All interviews were audio-recorded with consent, transcribed using Microsoft Teams’ transcription software, and pseudonymised prior to analysis. Although a formal sample size calculation was not performed, thematic saturation was assessed iteratively; by the final interviews, no substantively new themes were emerging, suggesting that the sample was sufficient for the study’s exploratory aims.

Interviews probed participants’ experiences using epidemic forecasts during the COVID-19 pandemic, including the types of forecasts they relied on, their perceived value, and the challenges encountered in their use. Participants were asked to reflect on the accessibility and usefulness of forecast metrics, the key policy questions forecasts addressed or failed to address, and the ways in which forecasts were evaluated before informing decisions. Additional questions explored perceived barriers to effective forecast use and solicited recommendations for improving forecast development, communication, and integration into policy (S1 Appendix B).

### Analysis

#### Survey data.

Survey responses were exported and analysed using R. Descriptive statistics (counts and percentages) were used to summarise quantitative responses, stratified by stakeholder type and World Bank income classification, with a primary comparison between economic development contexts. Denominators vary by question to account for item non-response and survey attrition. Given the exploratory design and relatively small subgroup sizes, the analysis prioritised pattern identification over hypothesis testing. Fisher’s exact tests were applied to key cross-tabulations to assess whether observed differences across income groups were consistent with chance; 95% Wilson confidence intervals are reported for selected proportions where cell counts permit. A binary sensitivity analysis (HIC/UMIC vs LMIC/LIC) was conducted alongside the four-group stratification.

All source code used for data analysis is available on GitHub (https://github.com/paulachristen/infectech_manuscript) [18].

#### Qualitative data.

Interview transcripts and responses to open-ended survey questions were analysed thematically using the Framework Method in NVivo (version 15). The Framework Method was selected for its suitability for applied policy research and its capacity to facilitate systematic comparison across predefined categories (e.g., country income groups and policy levels) while remaining open to emergent themes. Coding was conducted deductively, examining differences by the level of policy engagement (e.g., field-level, subnational, national) and economic development context.

To ensure consistency and reduce bias, data were double-coded independently by two researchers (PC and SvL), and coding discrepancies were resolved through discussion. Once sufficient inter-coder agreement was achieved (kappa > 0·75) [19], the remaining interviews and open-text responses were coded using the shared codebook (S1 Table). Themes were refined iteratively through memo writing, constant comparison across interviews, and collaborative team discussions. Initial kappa values for the double-coded subset ranged from 0.78 to 0.85 across major coding categories.

Quantitative and qualitative findings were integrated at the interpretation stage: survey results were used to identify patterns across settings, and interview data were then used to explain and contextualise these patterns, following a complementarity approach.

## Results

A total of 143 individuals enrolled in the online survey. Of these, 73% (105/143) reported involvement in COVID-19 policy dialogues at the national, sub-national, or field levels across 46 countries. Respondents reported working across multiple geographic levels: 20% (28/143) at the global level, 27% (38/143) at the regional level, 68% (97/143) at the national level, 36% (52/143) at the sub-national level, and 26% (37/143) at the field or community level. By country income classification, 13% (19/143) worked in low-income countries (LICs), 35% (50/143) in lower-middle-income countries (LMICs), 10% (14/143) in upper-middle-income countries (UMICs), and 23% (33/143) in high-income countries (HICs) during the COVID-19 pandemic. Further details on survey participation by section are provided in Fig A in S2 Appendix. In addition, thirteen participants took part in in-depth interviews, of which eleven were included in the analysis, having directly or indirectly engaged with epidemic forecasts (Table A in S2 Appendix). Participants were recruited through non-probability purposive and snowball sampling; the resulting sample was geographically concentrated, with the largest shares of respondents from the African Region, followed by the European and South-East Asia Regions. Subgroup sizes were uneven, particularly for UMICs (n = 14) and LICs (n = 19), which limits the precision of income-stratified comparisons.

Results are organised around three thematic domains - how forecasts were used, how they were communicated, and what shaped their uptake - integrating survey and interview findings within each domain to triangulate findings.

### How forecasts were used

#### Policy questions addressed by forecasts.

**Fig 1:**
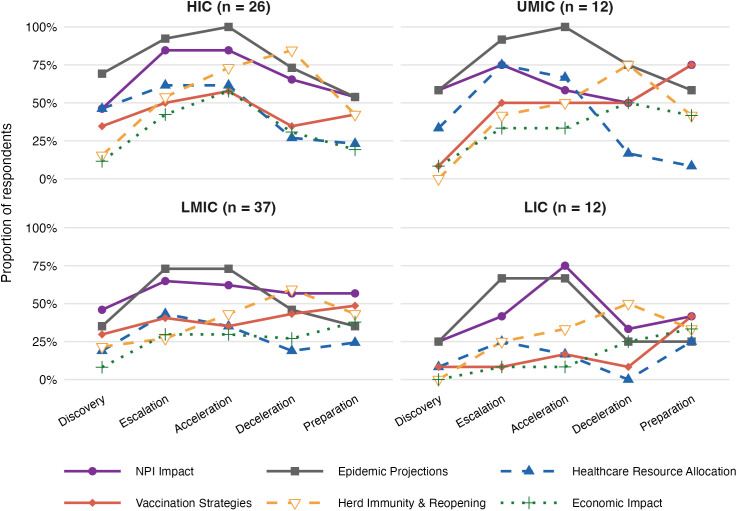
Forecast question categories across pandemic phases, by income group. For each phase, the proportion of respondents who selected at least one question from each category is shown. Sample sizes per group are shown in panel headers. HIC = high-income country; UMIC = upper-middle-income country; LMIC = lower-middle-income country; LIC = low-income country.

Epidemic forecasts were used to inform six key types of policy questions: NPIs and their impact, herd immunity and re-opening strategies, economic impact, healthcare resource allocation, vaccination strategies, and epidemic projections and modelling (Fig 1). These question themes shifted over the course of the pandemic, reflecting evolving policy priorities. Early forecasts were used to estimate epidemic size and model the effectiveness of NPIs. As case numbers accelerated, decision-makers sought to anticipate peaks and healthcare demands. In later stages, attention turned to the implications of lifting restrictions and behavioural change. This temporal pattern was consistent across income groups in the survey (Fig 1) and was corroborated in interviews, where respondents described a clear shift in the questions they brought to modellers:

“Before the pandemic, the policy solution was generally to crush the epidemic - reduce infections and hospitalizations to zero. After the pandemic began, the objective shifted: policymakers needed to understand not just what the epidemic curve would look like without intervention, but how different policies could shape that curve, balancing health outcomes with costs, education, and broader societal impacts.” (ID110)

#### Variation by setting and level.

At the global level, forecasts primarily informed high-level coordination and regional comparisons. A respondent from an international organization noted:

“A lot of our role was reporting upwards... giving a global overview of trends. It was very high level... more like: ‘In this region of the world, cases are increasing very quickly,’ or ‘cases are stable,’ or ‘we don’t really know what’s going on.’” (ID102)

At national and subnational levels, forecasts were used to anticipate epidemic size and timing, assess health system readiness, and guide contingency planning. One respondent explained how they “projected the disease pattern, mortality, and morbidity... calculated daily positivity rates, and forecast where we might land in two or three months” (ID105). In HIC and LMIC settings, forecasts also supported immediate planning, enabling policymakers to make informed decisions on resource allocation and capacity management, including whether to scale up testing, procure vaccines, and drugs, or reallocate hospital infrastructure (ID103, ID111). In Iceland, a HIC with a small, geographically concentrated population, a senior public health official (ID113) emphasized how local conditions shaped their use of forecasts. Without in-house modelling capacity, they relied on a university team to model epidemic trajectories and hospital admissions. These projections, communicated visually through epidemic curves, helped justify decisions to rapidly expand ICU capacity and informed public health regulations. At the same time, the respondent noted limitations in interpreting international models in their setting, where small case numbers and high day-to-day variation made local data difficult to work with. “We are so few,” they explained, “our numbers are always like this - like a sawtooth.” (ID113)

In LICs, forecasts also supported geographically and economically targeted responses. In Malawi, they guided the location of treatment centres and the delivery of support to vulnerable groups such as vendors and truck drivers during lockdowns (ID104). Geographic granularity enabled decision-makers to identify “townships that would be more affected than others” (ID104) and allocate resources accordingly. In Madagascar, daily forecasts were used to adjust ambulance deployment and manage patient flows based on projected demand (ID109).

#### Decision-relevant metrics.

**Fig 2:**
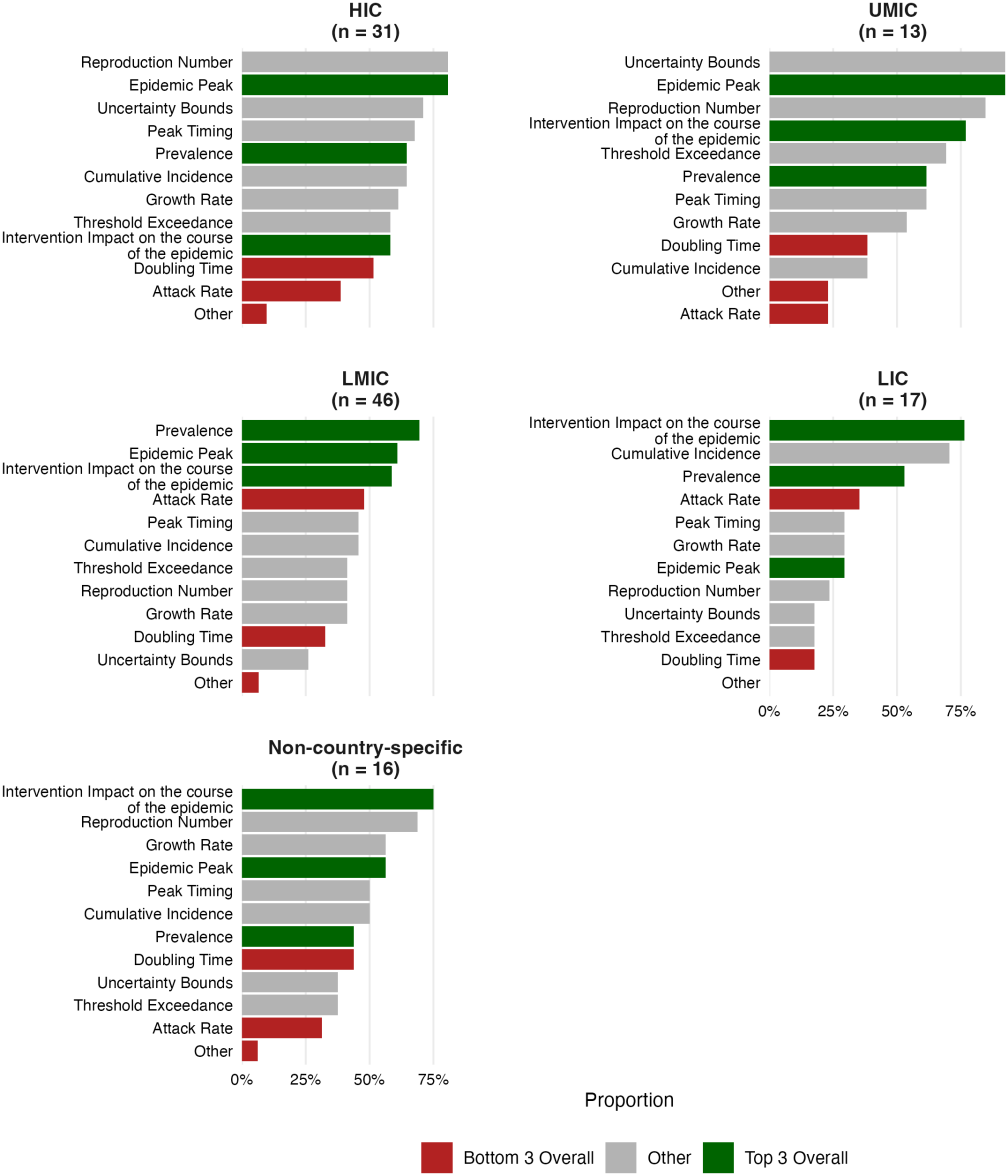
Forecast metrics valued by stakeholders, by income group. Bars show the proportion of respondents who identified each metric as important. Metrics are ranked within each panel by descending proportion. Green = top three overall; red = bottom three overall; grey = remaining metrics. Sample sizes per group are shown in panel headers. HIC = high-income country; UMIC = upper-middle-income country; LMIC = lower-middle-income country; LIC = low-income country.

In parallel with the evolution of policy questions, the specific metrics used to inform decisions shifted over time and across settings. Survey responses highlight that the most frequently communicated metrics included the projected impact of interventions (65%; 80/123), the epidemic peak (64%; 79/123), and the prevalence (62%; 76/123) (Fig 2).

However, usage patterns varied by setting. The size of the epidemic peak (e.g., the expected maximum number of daily hospital admissions) was among the top three metrics shared in all income settings except LICs, where the projected impact of interventions was most commonly communicated. The reproduction number was frequently cited in HICs, UMICs, and at the global and regional levels, but was rarely cited by respondents in LICs. Of the eleven metrics assessed, the sharpest income-group differences were observed for the reproduction number (Fisher’s exact test, p < 0·001) and uncertainty bounds (p < 0·001), both communicated far more frequently in HIC/UMIC than in LMIC/LIC settings; the epidemic peak also differed significantly (p = 0·02). Doubling time was among the least communicated metrics across most settings, with the exception of regional and global levels. Similarly, the attack rate ranked among the least used in LICs, but was more frequently reported in UMICs, where it was the fourth most commonly communicated metric. In Australia, focus initially centred on ICU occupancy, but shifted over time to more operational metrics like hospitalizations and workforce absenteeism, particularly during the Omicron wave (ID112).

### How forecasts were communicated

#### Formats and framing of forecasts.

**Fig 3:**
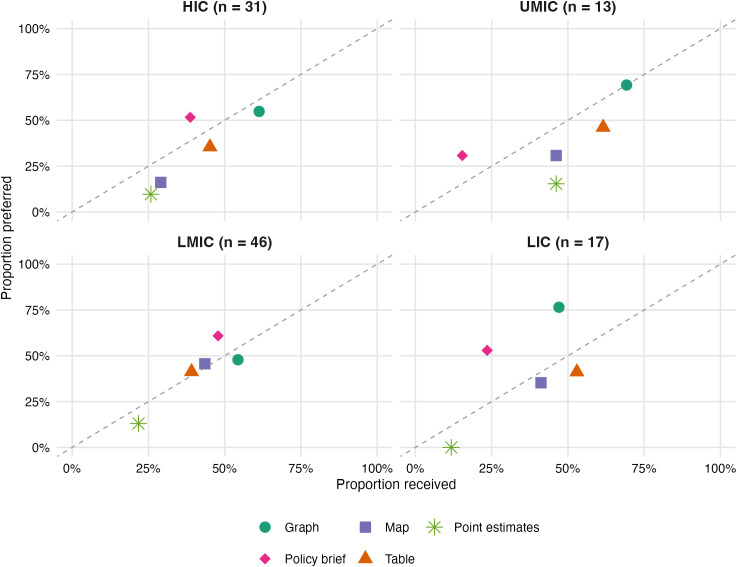
Format of epidemic forecast evidence received versus preferred, by income group. Each point represents one format type. Points above the diagonal indicate formats preferred more often than received; points below indicate formats received more often than preferred. Sample sizes per group are shown in panel headers.

Forecasts were received in a range of formats, with graphs (48%; 59/123), interactive dashboards (40%; 49/123), and scientific papers (39%; 47/123) among the most common (Fig 3). Across both survey and interview data, visual and concise outputs, particularly graphs, dashboards, and policy briefs, were consistently preferred over more technical formats such as point estimates or statistical intervals. These latter formats were often viewed as difficult to interpret or apply in real time. The survey confirmed this mismatch quantitatively: across all income groups, policy briefs and graphs were preferred more often than they were received, while scientific papers were received more often than preferred (Fig 3), a pattern that interview respondents attributed to the limited accessibility of technical publications in fast-moving policy environments. Receipt of scientific papers differed significantly by income group (Fisher’s exact test, p = 0·047), with higher proportions in HIC and UMIC settings.

Forecasts were most impactful when used to assess whether key policy thresholds had been crossed, supporting binary decisions such as whether to scale up testing or approve vaccine orders. Consistent with this, the survey showed that the projected impact of interventions was the most frequently valued metric overall (65%), ahead of more granular measures such as doubling time or attack rates (Fig 2). As one policymaker explained, “the issue is more about a binary decision, do I need to order more tests, do I need to sign off vaccines, rather than interpreting detailed point estimates” (ID110). This was echoed by another policymaker in Iceland (ID113).

Scenario-based presentations were highly valued across settings for conveying uncertainty and supporting adaptive decision-making. Respondents from Sweden (ID111), Zimbabwe (ID107), Lao PDR (ID103), and the UK (ID110) described using scenario comparisons to evaluate the implications of different actions, such as lockdowns, school closures, or changes in vaccine coverage.

#### Preferences for presentation and expert engagement.

Interactive dashboards received mixed feedback. Some users appreciated their ability to explore scenarios and monitor trends, particularly in countries with limited in-country modelling capacity (ID101). However, their utility was dependent on users’ time, digital skills, and familiarity with the tools, factors that were cited as barriers in LIC settings such as Malawi (ID104). This ambivalence was reflected in the survey, where interactive platforms were among the most commonly received formats (40%) but were not proportionally preferred, suggesting that access alone does not equate to utility without accompanying support.

**Fig 4:**
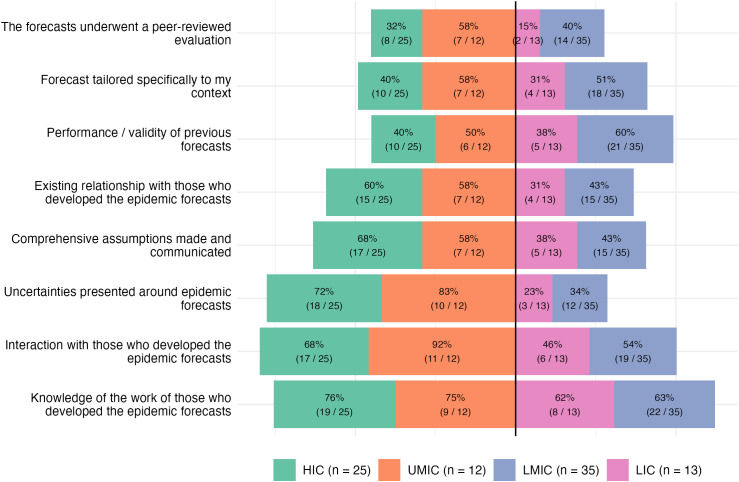
Factors contributing to confidence in epidemic forecasts, by income group. Bars show the proportion of respondents selecting each factor. HIC and UMIC extend leftward; LMIC and LIC extend rightward. Percentages and counts are labelled within bars. Sample sizes per group are shown in the legend. HIC = high-income country; UMIC = upper-middle-income country; LMIC = lower-middle-income country; LIC = low-income country.

Forecasts were most useful when accompanied by expert interpretation and interactive dialogue. Policymakers emphasized the value of real-time briefings that created space for questions and clarification, especially in LMICs and LICs where contextual understanding was crucial (ID110, ID107). In high-level policy settings, clarity and simplicity were also key. One respondent explained that decision-makers often preferred narrative framings over technical details, asking: “Is it going to be twice as bad, half as bad, or about the same?” (ID113). While dashboards were valued by some subnational actors, they were rarely used in senior policy meetings; visual summaries and concise briefings were favored. Without engagement, technically sound forecasts were often seen as less helpful or even confusing (ID101). The survey data corroborated this pattern: “interaction with those who developed the epidemic forecasts” was among the most frequently cited confidence factors across all income groups (Fig 4), reinforcing the qualitative emphasis on dialogue over static outputs. Notably, while valued universally, direct interaction with modellers was cited significantly more often by respondents in higher-income settings (Fisher’s exact test, p = 0·047), as were existing professional relationships with forecast developers (p = 0·044), suggesting that these forms of engagement may be less structurally available in lower-income contexts.

While scientific papers were valued for their credibility, they were not considered well-suited to decision-making in real time. One respondent remarked, “[scientific papers] shouldn’t be the main tool… but the proof of work is sometimes very helpful” (ID110).

Among survey respondents in HIC and UMIC, 72% (18/25) and 83% (10/12), respectively, identified the explicit presentation of uncertainty as a key confidence factor, compared to just 34% (12/35) in LMIC and 23% (3/13) in LIC (Fig 4). This difference was statistically significant when comparing higher-income (HIC/UMIC) with lower-income (LMIC/LIC) settings (Fisher’s exact test, p < 0·001). Interview data offered a possible explanation for this gradient: respondents in lower-income settings described prioritising source credibility and expert engagement over statistical uncertainty quantification as markers of trustworthiness. Qualitative descriptors such as “high certainty” or “low certainty” were considered more intuitive according to a respondent at an international organization (ID102).

### What shaped forecast uptake

#### Barriers to integration.

Barriers to the effective use of epidemic forecasts were most commonly rooted in foundational data limitations, issues of timing and delivery, challenges in interpretation, and misalignment with local contexts.

**Fig 5:**
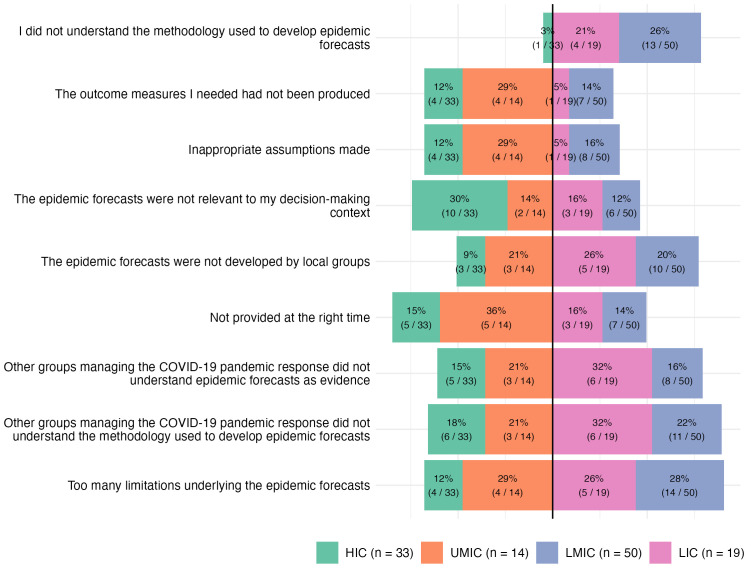
Barriers to using epidemic forecasts, by income group. Bars show the proportion of respondents selecting each barrier. Sample sizes per group are shown in the legend. HIC = high-income country; UMIC = upper-middle-income country; LMIC = lower-middle-income country; LIC = low-income country.

Unreliable, delayed, or incomplete data undermined the perceived credibility of forecasts; real-time decision-making was hindered when baseline data were missing or late. As one policymaker from Madagascar noted, “if the data had arrived on time, there wouldn’t have been forecasting problems” (ID109). Another added, “before the epidemic forecast, we need to have the baseline data” (ID105). Timing of forecast delivery also played a critical role. Forecasts that arrived too late, or without explanation, were less actionable. These issues were most frequently reported by respondents in UMICs (42%; 5/12) (Fig 5).

Several respondents noted that they or their colleagues did not fully understand the modelling approaches, which limited trust in and use of forecasts. Interview accounts illuminated the mechanisms behind these survey patterns: respondents described how limited exposure to quantitative modelling during training, combined with the novelty and speed of COVID-19 model production, left many policymakers unable to critically appraise forecast outputs or communicate them onward with confidence. Comprehension challenges among colleagues were particularly higher among LMIC and LIC respondents than LMIC, UMIC and HIC respondents: 32% (6/19) of LIC participants reported that others involved in the response did not understand the modelling evidence. Self-reported gaps in understanding were most common in LMICs (28%, 13/50) and LICs (21%, 4/19), compared to just 3% (1/33) in HICs and 0% in UMICs (Fig 5). This income-group difference in self-reported methodological understanding was statistically significant in the binary comparison (Fisher’s exact test, p < 0·01).

A lack of locally developed models was another barrier. 20% of LMIC respondents (10/50), 21% of UMIC respondents (3/14), and 26% of LIC respondents (5/19) cited the absence of locally adapted tools or capacity as a reason forecasts were underused or mistrusted. Notably, while the survey captured the prevalence of this barrier, interview data revealed an additional dimension not visible in the structured responses: several respondents described how externally produced models were not merely unused but actively resisted, because they were seen as disconnected from local surveillance realities and imposed without consultation (ID104, ID107). This divergence between passive non-use and active mistrust has implications for how capacity-building interventions are designed.

Table 1 synthesises the top-ranked metrics, confidence factors, and barriers by income group. Taken together, these patterns suggest a gradient in forecast engagement: respondents in higher-income settings prioritised statistical rigour and formal uncertainty quantification, whereas those in lower-income settings valued actionable, intervention-oriented outputs and identified comprehension and local capacity gaps as the primary barriers to use. Fisher’s exact tests confirmed that this gradient was statistically significant for several key items, including the use of reproduction numbers and uncertainty bounds as communicated metrics (both p < 0·001), explicit uncertainty presentation as a confidence factor (p < 0·001), and self-reported gaps in methodological understanding as a barrier (p < 0·01) (S2 Table).

**Table 1:** Survey findings by income group: Top-ranked forecast metrics, confidence factors, and barriers to use. Proportions reflect the percentage of respondents in each income group who selected each item.

Income group	Top valued metrics	Top confidence factors	Top barriers to use
**HIC** (n = 25)	• Epidemic peak (81%) • Reproduction number (81%) • Uncertainty bounds (71%)	• Knowledge of modellers’ prior work (76%) • Explicit uncertainty presentation (72%) • Direct interaction with modellers (68%) • Transparent assumptions (68%)	• Forecasts not relevant to context (30%) • Colleagues did not understand forecasts (18%) • Colleagues did not understand methodology (15%) • Untimely delivery (15%)
**UMIC** (n = 12)	• Epidemic peak (92%) • Uncertainty bounds (92%) • Reproduction number (85%)	• Direct interaction with modellers (92%) • Explicit uncertainty presentation (83%) • Knowledge of modellers’ prior work (75%)	• Untimely delivery (36%) • Needed outcome measures not produced (29%) • Inappropriate assumptions (29%) • Too many underlying limitations (29%)
**LMIC** (n = 35)	• Prevalence (70%) • Epidemic peak (61%) • Projected intervention impact (59%)	• Knowledge of modellers’ prior work (63%) • Track record of prior forecasts (60%) • Direct interaction with modellers (54%)	• Too many underlying limitations (28%) • Did not understand methodology (26%) • Colleagues did not understand methodology (22%)
**LIC** (n = 13)	• Projected intervention impact (76%) • Cumulative incidence (71%) • Prevalence (53%)	• Knowledge of modellers’ prior work (62%) • Direct interaction with modellers (46%) • Transparent assumptions (38%) • Track record of prior forecasts (38%)	• Colleagues did not understand methodology (32%) • Colleagues did not understand forecasts (32%) • Not developed by local groups (26%) • Too many underlying limitations (26%)

HIC = high-income countries; UMIC = upper-middle-income countries; LMIC = lower-middle-income countries; LIC = low-income countries.

## Discussion

To our knowledge, this study is among the few global, mixed-methods studies to systematically examine how epidemic forecasts were perceived, used, and communicated by public health decision-makers during the COVID-19 pandemic. Drawing on data from 46 countries across diverse economic contexts, the study addresses persistent gaps between the development of forecasts and their utility in decision-making in public health emergencies, particularly in resource-constrained settings. While forecasts were broadly recognised as valuable tools, their perceived utility depended on clear presentation, timely delivery, and alignment with operational needs.

Forecasts were most useful when tailored to the specific questions policymakers have. Although lists of potential policy questions have been proposed [20], these have rarely been developed collaboratively with end-users [21]. Our findings confirm that evidence needs are highly dynamic and context-specific. What policymakers require from forecasts is shaped by institutional roles, the stage of response, and existing decision-making structures [17]. Systematically documenting these needs, as our survey begins to do, offers a step toward structured anticipatory intelligence and reduces the reliance on modellers having to “guess” what is useful. The substantial variation in preferred metrics across income groups implies that efforts to improve forecast communication cannot rely on a single template; they must account for how local decision-making structures and technical capacity shape what different users find actionable.

Forecast tools are more effective when they present metrics relevant to the user’s decision-making context and align with cognitive preferences, rather than offering one-size-fits-all formats. Interactive, modular platforms that accommodate different user types (e.g., national vs. subnational decision-makers) and support adaptive visualisation can further enhance policy relevance. However, our survey also found that limited digital skills and unfamiliarity with such tools were barriers to forecast use. Tool development and user training should therefore be integrated into pandemic preparedness strategies, so that users are equipped to engage with these types of outputs before emergencies arise. Emerging evidence on forecast visualisation, including preferences for uncertainty display and layout clarity, can also inform design [22].

Beyond the barriers identified in our findings, emerging technological developments may help improve forecast-to-policy translation, though these were not directly examined in our study. Artificial intelligence (AI) methods, for instance, show promise for knowledge translation: AI-powered summarisation could convert complex model outputs into clear, policy-relevant insights. Separately, advances in time-series foundation models and ensemble forecasting may improve the speed and robustness of predictions, even in data-sparse settings [23,24]. These tools are especially valuable in low-resource contexts where forecasts are developed externally but used locally [25]. Open-source packages with templates for uncertainty bands, scenario plots, and policy triggers could help bridge communication gaps between modellers and decision-makers. Without such tools, poorly conveyed uncertainty risks eroding trust and reducing forecast utility [26]. While technical improvements are critical, they rely on strong foundations. With global health funding cuts, sustaining core data infrastructure and custodianship is essential. Reliable, timely data forms the backbone of forecasts [9], yet in many settings, especially lower income settings, data systems remain tied to short-term projects and international aid, making them vulnerable to collapse when funding ends [27]. Without stable investment, data collection, quality assurance, and institutional memory erode, undermining even the most sophisticated models [28]. When full-scale modelling efforts are not feasible due to funding constraints, protecting essential data systems is vital for ensuring relevant evidence during future public health crises.

However, having robust data is only part of the solution. We found that confidence in forecasts often rested less on statistical sophistication and more on interpersonal familiarity, institutional credibility, and shared understanding, particularly in LICs and LMICs. Ensuring that data is translated into timely, decision-relevant insights will still require formal mechanisms, such as data-sharing agreements, memoranda of understanding, and embedding modellers within surveillance units or emergency response centres. These approaches can foster trusted collaboration. Furthermore, when modellers are closely engaged with operational surveillance teams, they become more attuned to data limitations and better positioned to shape what is collected and how it is used [29].

Taken together, the quantitative and qualitative findings converge on a central insight: the policy utility of epidemic forecasts is shaped not primarily by their technical sophistication, but by the ecosystem in which they are produced, communicated, and received. Survey data revealed systematic variation across income groups in preferred metrics, formats, and confidence factors, while interviews illuminated the relational and institutional mechanisms underlying these patterns. In higher-income settings, forecast utility was facilitated by in-country modelling capacity and established science-policy interfaces, whereas in lower-income settings, utility depended more heavily on interpersonal trust, contextual adaptation, and the availability of expert intermediaries. This complementarity between the quantitative breadth and qualitative depth of our findings underscores that improving forecast-policy alignment will require simultaneous investment in technical tools, institutional relationships, and local capacity.

This study has several limitations. First, it is subject to recall bias, as participants were asked to reflect on decisions and interactions that occurred during the COVID-19 pandemic. Future research could mitigate this by embedding similar assessments within real-time epidemic responses or simulation exercises, such as the Polaris Exercise conducted by WHO to test the Global Health Emergency Corps. Second, non-response bias is likely. Key workers, who experienced considerable psychological strain during the pandemic [30], may have been less willing to revisit those experiences. Furthermore, while we focused on individuals involved in feeding evidence into political decision-making, our findings may not fully capture the perspectives or priorities of politicians themselves. Future research should endeavour to include political decision-makers directly. However, accessing their perspectives remains challenging due to the small number of individuals at higher levels of decision-making, their limited availability, and the protective gatekeeping that often surrounds them [31]. Third, although the survey achieved broad geographic reach, attrition over its course (Fig A in S2 Appendix) may have weakened the representativeness of responses in later sections. Further, the study relied on self-reported involvement in policy dialogue, which we were unable to independently verify; not all participants may have had the same level of engagement in decision-making during the pandemic, and this self-selection criterion risks misclassification – for example, individuals with peripheral advisory roles may have interpreted “policy dialogue” differently from those directly involved in decision-making. Fifth, the convenience sampling strategy likely over-represents individuals connected to modelling and academic networks, which may limit generalisability to broader policy communities, particularly in settings where such networks are less established. Sixth, the survey remained open for six months (September 2024–March 2025), during which post-pandemic policy contexts continued to evolve; respondents completing the survey later may have had different retrospective assessments than those responding earlier, introducing potential temporal bias that we were unable to assess given the anonymous design. These biases should be kept in mind when interpreting the descriptive patterns reported, particularly the comparisons across income groups, which are based on small non-representative subsamples.

## Conclusion

Epidemic forecasting will influence public health decision-making only when it is embedded within the institutional, political, and operational contexts in which decisions are made. This study demonstrates that the utility of forecasts depends not only on their technical quality, but on their contextual relevance, clarity of communication, and alignment with specific policy choices.

Our findings show that forecasts were most actionable when they supported binary or scenario-based decisions and were accompanied by expert interpretation. However, substantial disparities in forecast access, comprehension, and trust persist across economic settings. Lower-income countries face compounding constraints related to data infrastructure, modelling capacity, and institutional linkages between analysts and policymakers.

Strengthening the policy impact of epidemic forecasting therefore requires action across the preparedness cycle. Forecasting must be institutionalised as part of decision-support systems rather than treated solely as a technical modelling exercise. Preparedness efforts should clarify the types of decisions forecasts are expected to inform, enabling data systems, modelling design, and communication strategies to be structured accordingly. Investment in modular, user-oriented communication tools, embedded modelling capacity within response structures, and sustained health data systems will be essential. Crucially, both tool development and user training must occur during periods of stability so that forecasts can be rapidly understood, trusted, and deployed when emergencies arise.

Emergency conditions demand a rethinking of how evidence is produced, disseminated, and interpreted. While the principles of effective knowledge translation – timeliness, accessibility, interpretability, and transparency – are well-established [32,33], they must be retooled for contexts where decisions are time-sensitive, information is partial, and the policy process is nonlinear. Operationalising these principles remains an urgent task for researchers, funders, and international institutions alike, building on efforts by groups such as WHO and others to ensure that modelling serves not just the advancement of science, but public health.

## Supporting information

S1 Appendix Survey instrument and semi-structured interview guide. Contains the full survey questionnaire and interview guide used in the mixed-methods study exploring the use, communication, and policy relevance of epidemic forecasts for policymakers.
(DOCX)

S2 Appendix Study participant characteristics and survey dropout analysis. Includes survey dropout rates by respondent income classification and characteristics of qualitative interview participants, including geographic scope, organization type, and participant profiles.
(DOCX)

S1 Table Qualitative codebook used for thematic analysis. Provides the hierarchical coding framework, including parent themes, subcodes, and descriptions, developed iteratively for the analysis of semi-structured interviews.
(DOCX)

S2 Table Fisher’s exact tests for survey items by World Bank income classification. Presents subgroup analyses comparing survey responses across low-, lower-middle-, upper-middle- and high-income country groups, including reported metrics, forecast formats, confidence factors, and barriers to forecast use.
(DOCX)
