# Supplementary material for: Enhancing epidemic forecast usability for policymakers: A global mixed-methods study
Source: PLOS Glob Public Health. 2026 Jun 4;6(6):e0006519. doi: 10.1371/journal.pgph.0006519 (PMC13235937; doi:10.1371/journal.pgph.0006519)
Supplement: S1 Appendix — Contains the full survey questionnaire and interview guide used in the mixed-methods study exploring the use, communication, and policy relevance of epidemic forecasts for policymakers. (DOCX) [file pgph.0006519.s001.docx]

Enhancing Epidemic Forecast Usability for Policymakers: A Global Mixed-Methods Study

S1 Appendix

## Survey

## Interview guide

Semi-structured interview questions

**Enhancing Epidemic Forecast Usability for Policymakers: A Global Mixed-Methods Study**

*Principal Investigator: Oliver J Watson*

*Co-investigators: Paula Christen, Loice Achieng, Jeanette Dawa, Thumbi Mwangi, Charlie Whittaker, Lilith Whittles, Njoki Kimani, Maria Veras*

1. **Introduction**
   1. Interviewer introduces themselves and their engagement in this work.
   2. Explain background of research.
   3. Aims of research:

Our goal is to bridge the gap between scientific forecasts and practical decision-making, especially in resource-constrained settings.

Your experiences will help us:

- Identify unmet needs related to epidemic forecasting.
- Develop user-friendly tools to evaluate and interpret forecasts for policymakers.

1. Explain what to expect in the interview.
2. Shortly reiterate information provided in survey.

**Round 1**

1. **Interview**
   1. **Section 1 – Reflections on the role of epidemic forecasts during the COVID-19 pandemic response**
      1. How do you describe the role of epidemic forecasts during the COVID-19 pandemic response?
      2. Which types of epidemic forecasts (e.g., short-term case projections, hospital bed occupancy predictions, long-term scenario modeling) proved most valuable for decision-making? Why?
      3. What were the biggest challenges in using epidemic forecasts effectively during the rapidly evolving COVID-19 pandemic? Were there issues with data quality, model accuracy, or communication of results?
      4. How can we improve the development, communication, and utilization of epidemic forecasts to better prepare for future outbreaks?

1. **Section 2 – Reiteration of findings from survey**

So far, we have received xxx survey responses. Based on a preliminary analysis, we now better understand relevant metrics, questions, methods and requirements for evaluations of epidemic forecasts as well as barriers to using epidemic forecasts.

In the next section, we would like to probe these findings to understand whether these preliminary findings resonate with you and speak to your needs.

1. **Metrics: To understand the most accessible format of epidemic forecasts in public health decision-making.**

Confirmation:

- Our survey suggests that [xxx] was the most accessible and useful format in which epidemic forecasts were presented/made available. Does this align with your experience? Why? Why not?
- Our survey suggests that [xxx] were commonly communicated to supervisors or in meetings. Does this align with your experience? Why? Why not?

Value: Were these the most valuable types of forecast metrics for you, or were there others you found more useful? Why?

1. **Questions: To identify the key policy questions and decisions that epidemic forecasts helped address.**

Relevance: Our survey identified the following as the top questions addressed by forecasts.

[show and read printed list of key policy questions]

Did these questions align with your primary concerns during the pandemic?

Unmet Needs: Were there any critical policy questions that epidemic forecasts didn't address, but that you would have found valuable?

Priority: If you could only have forecasts for a limited number of questions, which would you prioritize and why?

1. **Evaluation: To learn how epidemic forecasts were evaluated (if at all) before being used in decision-making.**

Practices: Our survey found that [summarize evaluation methods] were the most common ways forecasts were evaluated. Does this reflect your approach?

Satisfaction: Were they sufficient to assess forecast accuracy and reliability?

Ideal Evaluation: In an ideal scenario, how would you like epidemic forecasts to be evaluated before being used in decision-making?

1. **Barriers: To uncover the main challenges that hindered the use and usefulness of epidemic forecasts for policymakers.**

Top Barriers: Our survey highlighted [list top barriers] as the main challenges to using forecasts effectively. Do these resonate with your experience?

Solutions: What steps could be taken to overcome these barriers and make forecasts more useful for policymakers like yourself?

**Wrap-Up:** Thank the participant for their time and valuable feedback.
