# Supplementary material for: Enhancing epidemic forecast usability for policymakers: A global mixed-methods study
Source: PLOS Glob Public Health. 2026 Jun 4;6(6):e0006519. doi: 10.1371/journal.pgph.0006519 (PMC13235937; doi:10.1371/journal.pgph.0006519)
Supplement: S2 Appendix — Includes survey dropout rates by respondent income classification and characteristics of qualitative interview participants, including geographic scope, organization type, and participant profiles. (DOCX) [file pgph.0006519.s002.docx]

Enhancing Epidemic Forecast Usability for Policymakers: A Global Mixed-Methods Study

S2 Appendix

# Study Participants


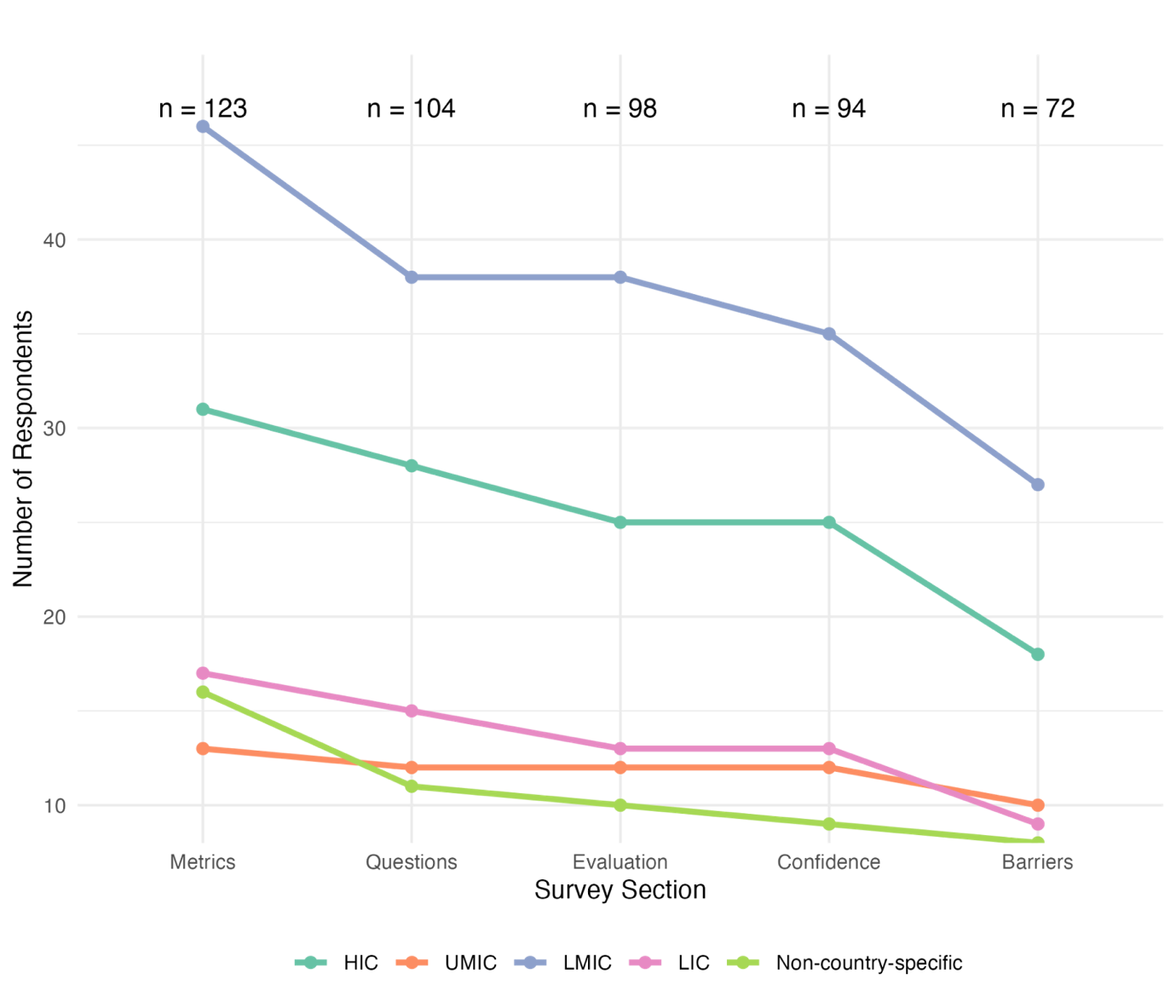


***Fig A. Survey dropout rates by section and overall sample group.***

*The figure shows the percentage of respondents who dropped out of the survey at each section: Metrics, Questions, Evaluation, Confidence, and Barriers. Dropout rates are presented respondents’ country income category, classified using the World Bank categorization (2023): HIC = high-income country; UMIC = upper-middle-income country; LMIC = lower-middle-income country; LIC = low-income country.*

**Table A. Characteristics of key informants participating in qualitative interviews**. The table summarizes the WHO region (AFRO = African Region, AMRO = Region of the Americas, EMRO = Eastern Mediterranean Region, EURO = European Region, WPRO = Western Pacific Region; "Global" denotes participants with a multilateral or global mandate spanning multiple regions) and country income classification (LIC = low-income country, LMIC = lower-middle-income country, HIC = high-income country)

| **ID** | **WHO region** | **Primary geographic scope of work** | **Country income group** | **Type of organization** | **Profile** |
| --- | --- | --- | --- | --- | --- |
| 101 | EURO | Regional (Europe) |  | International organization | A senior infectious disease modeller at a regional public health agency during the COVID-19 pandemic. The participant led epidemic modelling efforts, coordinated cross-country forecast use, and supported high-level decision-making across multiple countries in the region. |
| 102 | Global | Global |  | International organization | An infectious disease modeller based at a multilateral public health agency with a global mandate. During the COVID-19 pandemic, they contributed to both high-level situational assessments and operational forecasting support for individual countries. Their work focused on producing in-house analyses using official data, and they emphasized the importance of timely, context-sensitive modelling outputs and clear communication of uncertainty to support decision-making. |
| 103 | WPRO | Lao PDR | LMIC | Government agency | A senior public health official with extensive experience in infectious disease control and emergency preparedness. They have held leadership roles within a national health ministry and were involved in managing responses to previous respiratory outbreaks. During the early stages of the COVID-19 pandemic, they coordinated national efforts and collaborated with international partners to develop scenario-based forecasts. These forecasts played a key role in informing preparedness planning and high-level policy decisions such as resource distribution and border control strategies. |
| 104 | AFRO | Malawi | LIC | Government agency | A senior official at a national ministry of health, with a portfolio in digital health and data management. With a background in medicine and health systems strengthening, they were involved in the national COVID-19 response and worked closely with modelling teams to inform planning and resource allocation. |
| 105 | EMRO | Pakistan | LMIC | Government agency | A senior government epidemiologist with leadership responsibilities in outbreak response coordination. With training in field epidemiology and global health security, they played a key role in coordinating COVID-19 screening, response, and risk assessment. |
| 106 | AMRO | Regional (Americas) |  | International organization | A regional public health official with responsibilities in emergency preparedness and outbreak response. During the COVID-19 pandemic, they used epidemic forecasts to inform supply planning and assess the potential impact of different public health strategies. |
| 107 | AFRO | Zimbabwe | LMIC | Hospital / Clinic / Other healthcare provider | A public health leader with experience in infectious disease modelling and policy advising. They contributed to coordinating modelling inputs during the COVID-19 pandemic and collaborated with international partners to produce scenario-based forecasts. These forecasts informed government decision-making on public health measures such as lockdowns, school closures, and vaccination strategies. |
| 108 | AFRO | Kenya | LMIC | NGO | A social development practitioner and policy advisor involved in coordinating community-level COVID-19 response activities, including risk communication, service linkages, and support for vulnerable populations. |
| 109 | AFRO | Madagascar | LMIC | Government agency | A subnational public health official working during the COVID-19 pandemic. Their responsibilities included overseeing hygiene, sanitation, and patient logistics across health facilities in an urban area. They used short-term forecasts from a national health operations centre to inform daily decisions on patient placement, ambulance routing, and resource allocation. |
| 110 | EURO | UK | HIC | Government agency | A senior modeller and public health advisor with responsibilities spanning infectious disease modelling, analytics, and policy engagement during the COVID-19 pandemic. They contributed to national forecasts and collaborated with academic institutions to inform real-time decisions on interventions, healthcare capacity, and broader societal trade-offs. |
| 111 | EURO | Sweden | HIC | Government agency | A senior public health official with a background in infectious disease epidemiology and leadership experience in outbreak response. During the COVID-19 pandemic, they contributed to the development and communication of national epidemic forecasts, focusing on short-term hospital and ICU demand. Forecasts were presented to government and healthcare system stakeholders using scenario-based graphs to communicate uncertainty. |
| 112 | WPRO | Australia | HIC | University | An academic expert in epidemiology who contributed to a national infectious disease modelling consortium during the COVID-19 pandemic. They contributed modelling analyses to policy advisory groups, supporting public health decisions such as setting vaccine coverage targets for reopening, designing outbreak response strategies, and informing workforce planning during periods of high transmission. |
| 113 | EURO | Iceland | HIC | Government agency | A senior official within a national public health agency. With a background in clinical and quantitative training, they moved into public health leadership shortly before the COVID-19 pandemic. During the pandemic, they were involved in national coordination, policymaking, and the implementation of emergency health measures, including border controls, digital contact tracing, and public guidance. |
