# Supplementary material for: Enhancing epidemic forecast usability for policymakers: A global mixed-methods study
Source: PLOS Glob Public Health. 2026 Jun 4;6(6):e0006519. doi: 10.1371/journal.pgph.0006519 (PMC13235937; doi:10.1371/journal.pgph.0006519)
Supplement: S1 Table — Provides the hierarchical coding framework, including parent themes, subcodes, and descriptions, developed iteratively for the analysis of semi-structured interviews. (DOCX) [file pgph.0006519.s003.docx]

Enhancing Epidemic Forecast Usability for Policymakers: A Global Mixed-Methods Study

S1 Table

**Qualitative codebook: thematic codes and subcodes used in the analysis of semi-structured interviews.** Codes are organised hierarchically under four parent themes — challenges encountered in forecast use, evidence communicated to decision-makers, format and presentation of forecasts, and solutions or enablers identified by respondents. Descriptions summarise the scope of each code. The codebook was developed iteratively through a combination of deductive framing (informed by the interview guide) and inductive refinement during coding.

| **Name** | **Description** |
| --- | --- |
| Challenges |  |
| Accessibility | Measures to control access and prevent misuse of forecasts |
| Capacity | Resources or ability to conduct forecasting (e.g., personnel, tools) |
| Communication | Methods or strategies for sharing forecast results |
| Evidence user | Stakeholders or audiences interpreting and using forecast evidence |
| Limitations | Constraints or weaknesses in forecasts communicated to policymakers |
| Context | Situational factors influencing forecast relevance or application |
| Data availability |  |
| Data management system |  |
| Decisionmaking process | Steps or methods by which forecasts inform choices |
| Mental health | Psychological impacts or considerations related to forecasts |
| Multiple models | Use of several models for comparison or robustness |
| Policy decisions | Alignment of forecasts with actionable policy recommendations |
| Questions | Specific inquiries about policymaker needs or forecast applications |
| Understanding policymakers | Insight into the decision-making needs and priorities of policymakers |
| Responsibilities | Duties or roles of individuals or groups in forecasting |
| Sentiment | Emotional tone or public perception influencing or influenced by forecasts |
| Standardization | Adoption of uniform methods or formats in forecasting |
| Timeframe | Time constraints or planning horizons for forecasting activities |
| Modellers' capacity | Limitations in time or resources available to forecasting experts |
| UI UX | User interface and experience design for forecast presentation |
| Uncertainty | Sources or implications of unpredictability in forecasts |
| Evaluation | Assessment of forecast quality, accuracy, or utility |
| Assumptions | Underlying premises or conditions assumed in the model |
| Calibration | Process of adjusting model parameters to fit observed data |
| Comparison | Analysis of differences or similarities between models or forecasts |
| Ensemble models | Combined predictions from multiple models for consensus |
| Validation of forecasts | Verification of forecast accuracy against real-world outcomes |
| Evidence recipient |  |
| Forecast types (outcomes) | Types of predicted results or metrics being forecasted |
| Cases | Number or incidence of specific instances (e.g., infections) |
| Deaths | Number of fatalities associated with an event or condition |
| Economics | Economic outcomes or costs related to the forecast |
| Hospital demand | Predicted need for hospital resources (e.g., admissions, ICU beds) |
| Scenarios | Hypothetical situations or conditions modeled in forecasts |
| Uncertainty | Degree of unpredictability or variability in forecast results |
| Wave dynamics | Patterns or trends in event progression (e.g., epidemic waves) |
| Format |  |
| Diversity | Variety of approaches, outputs, or perspectives in forecasting |
| Graphs | Visual representations of data using charts or plots |
| Interactive | Features allowing user interaction with forecast outputs (e.g., dashboards) |
| Layout design | Visual organization using color-coded indicators (e.g., traffic light system green, yellow, red) for risk levels |
| Pdf report | Static document summarizing forecast results in PDF format |
| Raw data | Unprocessed data underlying the forecasts |
| Policymaking context | Policymaking context specific information |
| Questions | Topics or inquiries guiding the analysis or forecast |
| Economics | Economic implications or considerations |
| Healthcare capacity | Ability of healthcare systems to meet demand (e.g., beds, staff, equipment) |
| Healthcare demand | Demand for healthcare capacity |
| Interventions | Actions or strategies implemented to influence outcomes |
| Economics | Economic analysis of intervention costs, benefits, or impacts |
| Effectiveness | Measure of how well interventions achieve intended outcomes |
| Impact | Projected impact |
| Non-pharmaceutical interventions | Projected impact of non-pharmaceutical interventions |
| Policy options | Projected impact of policy, e.g., lockdown, mobility |
| Surveillance testing | Projected impact of surveillance / testing |
| Vaccines | Projected impact of vaccines |
| Variants | Projected impact of variants |
| Need for | Justification or requirement for implementing interventions |
| NPIs | Projected need for NPIs |
| Surveillance testing | Projected need for surveillance / testing |
| Vaccines | Projected need for vaccines |
| Outbreaks size magnitude | Scale or extent of an event (e.g., disease outbreak) |
| Risk | Likelihood and consequence of adverse outcomes |
| Severity | Degree of impact or intensity of an event or outcome |
| Timeframe | Duration or period over which forecasts or evaluations are made |
| Solutions | Proposed methods or approaches to address challenges |
| Acceptability | Degree to which forecast outputs are agreeable to stakeholders |
| Format | Format of relevance in the perspective of the recipient |
| Accessibility | Ease of access to forecast outputs for intended users; transparency of authorship |
| Applicability | Extent to which forecasts can be applied across different scenarios |
| Consistency | Uniformity in forecast presentation or methodology across outputs |
| Context | Specific setting or circumstances for which forecasts are tailored |
| Diversity | Inclusion of varied groups or perspectives in forecast development |
| Funding | Financial resources supporting forecasting efforts |
| Guidelines framework standardization | Established rules or structures for consistent forecasting |
