# Supplementary material for: Enhancing epidemic forecast usability for policymakers: A global mixed-methods study
Source: PLOS Glob Public Health. 2026 Jun 4;6(6):e0006519. doi: 10.1371/journal.pgph.0006519 (PMC13235937; doi:10.1371/journal.pgph.0006519)
Supplement: S2 Table — Presents subgroup analyses comparing survey responses across low-, lower-middle-, upper-middle- and high-income country groups, including reported metrics, forecast formats, confidence factors, and barriers to forecast use. (DOCX) [file pgph.0006519.s004.docx]

Enhancing Epidemic Forecast Usability for Policymakers: A Global Mixed-Methods Study

S2 Table

**Fisher’s exact tests for survey items by World Bank income classification.** Proportions show the percentage of respondents selecting each item, with counts in parentheses. Denominators vary by question due to item non-response. The 4-group column reports Fisher’s exact test p-values across all four income categories (LIC, LMIC, UMIC, HIC); the binary column reports the p-value for the collapsed comparison HIC/UMIC vs LMIC/LIC. Income groups follow the World Bank 2023 classification.

| **Domain** | **Item** | **LIC**  **% (n/N)** | **LMIC**  **% (n/N)** | **UMIC**  **% (n/N)** | **HIC**  **% (n/N)** | **p-value**  **(4-group)** | **p-value**  **(binary)** |
| --- | --- | --- | --- | --- | --- | --- | --- |
| **Metrics communicated** | Epidemic peak | 30.8 (4/13) | 62.5 (25/40) | 90.0 (9/10) | 75.0 (18/24) | 0.018 | 0.023 |
|  | Threshold exceedance | 23.1 (3/13) | 45.0 (18/40) | 60.0 (6/10) | 58.3 (14/24) | 0.183 | 0.123 |
|  | Uncertainty bounds | 23.1 (3/13) | 27.5 (11/40) | 90.0 (9/10) | 70.8 (17/24) | <0.001 | <0.001 |
|  | Peak timing | 30.8 (4/13) | 45.0 (18/40) | 50.0 (5/10) | 66.7 (16/24) | 0.182 | 0.081 |
|  | Cumulative incidence | 76.9 (10/13) | 47.5 (19/40) | 40.0 (4/10) | 66.7 (16/24) | 0.141 | 0.825 |
|  | Prevalence | 53.8 (7/13) | 67.5 (27/40) | 70.0 (7/10) | 62.5 (15/24) | 0.835 | 1.000 |
|  | Reproduction number | 30.8 (4/13) | 40.0 (16/40) | 90.0 (9/10) | 87.5 (21/24) | <0.001 | <0.001 |
|  | Doubling time | 23.1 (3/13) | 30.0 (12/40) | 40.0 (4/10) | 54.2 (13/24) | 0.179 | 0.067 |
|  | Growth rate | 30.8 (4/13) | 42.5 (17/40) | 40.0 (4/10) | 62.5 (15/24) | 0.260 | 0.186 |
|  | Attack rate | 30.8 (4/13) | 45.0 (18/40) | 20.0 (2/10) | 45.8 (11/24) | 0.441 | 0.825 |
|  | Intervention impact | 69.2 (9/13) | 45.0 (18/40) | 80.0 (8/10) | 54.2 (13/24) | 0.169 | 0.380 |
| **Format received** | Map | 53.8 (7/13) | 52.5 (21/40) | 60.0 (6/10) | 37.5 (9/24) | 0.584 | 0.512 |
|  | Table | 69.2 (9/13) | 47.5 (19/40) | 80.0 (8/10) | 58.3 (14/24) | 0.235 | 0.374 |
|  | Graph | 61.5 (8/13) | 62.5 (25/40) | 90.0 (9/10) | 79.2 (19/24) | 0.240 | 0.057 |
|  | Point estimates | 15.4 (2/13) | 25.0 (10/40) | 60.0 (6/10) | 33.3 (8/24) | 0.120 | 0.093 |
|  | Interactive platform | 53.8 (7/13) | 50.0 (20/40) | 80.0 (8/10) | 41.7 (10/24) | 0.236 | 1.000 |
|  | Policy brief | 30.8 (4/13) | 55.0 (22/40) | 20.0 (2/10) | 50.0 (12/24) | 0.157 | 0.514 |
|  | Scientific paper | 23.1 (3/13) | 47.5 (19/40) | 80.0 (8/10) | 58.3 (14/24) | 0.047 | 0.048 |
| **Confidence factors†** | Interaction with developers | 46.2 (6/13) | 54.3 (19/35) | 91.7 (11/12) | 68.0 (17/25) | 0.021 | 0.047 |
|  | Peer-reviewed evaluation | 15.4 (2/13) | 28.6 (10/35) | 50.0 (6/12) | 36.0 (9/25) | 0.072 | 1.000 |
|  | Existing relationship | 7.7 (1/13) | 22.9 (8/35) | 25.0 (3/12) | 36.0 (9/25) | 0.094 | 0.044 |
|  | Knowledge of developers’ work | 61.5 (8/13) | 62.9 (22/35) | 75.0 (9/12) | 76.0 (19/25) | 0.153 | 0.078 |
|  | Uncertainties presented | 23.1 (3/13) | 34.3 (12/35) | 83.3 (10/12) | 72.0 (18/25) | 0.003 | <0.001 |
|  | Comprehensive assumptions | 38.5 (5/13) | 37.1 (13/35) | 58.3 (7/12) | 68.0 (17/25) | 0.073 | 0.016 |
|  | Forecast tailored to context | 15.4 (2/13) | 34.3 (12/35) | 25.0 (3/12) | 20.0 (5/25) | 0.135 | 0.645 |
|  | Performance of previous forecasts | 38.5 (5/13) | 60.0 (21/35) | 41.7 (5/12) | 48.0 (12/25) | 0.500 | 1.000 |
| **Barriers to use** | Did not understand forecasts | 0.0 (0/13) | 7.5 (3/40) | 0.0 (0/10) | 0.0 (0/24) | 0.516 | 0.278 |
|  | Others did not understand forecasts | 46.2 (6/13) | 15.0 (6/40) | 20.0 (2/10) | 20.8 (5/24) | 0.154 | 1.000 |
|  | Did not understand methodology | 15.4 (2/13) | 20.0 (8/40) | 0.0 (0/10) | 0.0 (0/24) | 0.048 | 0.006 |
|  | Others did not understand methodology | 38.5 (5/13) | 20.0 (8/40) | 20.0 (2/10) | 25.0 (6/24) | 0.595 | 1.000 |
|  | Not relevant to context | 23.1 (3/13) | 15.0 (6/40) | 10.0 (1/10) | 33.3 (8/24) | 0.320 | 0.295 |
|  | Outcome measures not produced | 7.7 (1/13) | 15.0 (6/40) | 40.0 (4/10) | 16.7 (4/24) | 0.239 | 0.252 |
|  | Not provided at right time | 7.7 (1/13) | 20.0 (8/40) | 40.0 (4/10) | 12.5 (3/24) | 0.233 | 0.779 |
|  | Not developed by local groups | 23.1 (3/13) | 20.0 (8/40) | 30.0 (3/10) | 12.5 (3/24) | 0.643 | 0.788 |
|  | Inappropriate assumptions | 0.0 (0/13) | 17.5 (7/40) | 30.0 (3/10) | 8.3 (2/24) | 0.135 | 1.000 |
|  | Too many limitations | 15.4 (2/13) | 27.5 (11/40) | 30.0 (3/10) | 8.3 (2/24) | 0.232 | 0.416 |

*HIC = high-income countries; UMIC = upper-middle-income countries; LMIC = lower-middle-income countries; LIC = low-income countries.*

*† Denominators for confidence factors (Q24) differ from other domains as this question was asked only of respondents who reported using forecasts in policy dialogues (n = 85).*
